# Facile preparation of Ti_3_C_2_T_x_ sheets by selectively etching in a H_2_SO_4_/H_2_O_2_ mixture

**DOI:** 10.3389/fchem.2022.962528

**Published:** 2022-10-19

**Authors:** Zhengshan Tian, Hao Tian, Kesheng Cao, Suzhen Bai, Qinlong Peng, Yabo Wang, Qiuxiang Zhu

**Affiliations:** ^1^ School of Chemistry and Environmental Engineering, Henan Key Laboratory of Germplasm Innovation and Utilization of Eco-economic Woody Plant, Pingdingshan University, Pingdingshan, China; ^2^ School of Pharmacy, Henan University of Chinese Medicine, Zhengzhou, China; ^3^ College of Information and Electronic Engineering, Hunan City University, Yiyang, China

**Keywords:** Ti_3_AlC_2_ powders, Ti_3_C_2_T_x_ sheets, H_2_SO_4_/H_2_O_2_ mixture, selective etching, HF-containing etchants

## Abstract

MXenes and MXene-based composite materials have potential applications in a wide range of areas due to their unique physical and chemical characteristics. At present, it is still a major challenge to develop a simple, safe, and efficient route to prepare MXenes without using fluorinated etchants. Herein, we design a facile method to prepare Ti_3_C_2_T_x_ MXene sheets by selectively etching Ti_3_AlC_2_ powders in an aqueous diluted H_2_SO_4_ solution with H_2_O_2_ as an oxidant. In a system of H_2_SO_4_ and H_2_O_2_, an aqueous H_2_SO_4_ solution with a concentration of 6 mol/L is a strongly acidic medium with no volatility, and 30% H_2_O_2_ acts as a strong green oxidizer without harmful by-products. The experimental process is safe and convenient to conduct in a beaker under a water bath of 40°C. The etching process can be completed in 1 h under the air atmosphere conditions. The experimental results confirmed that the etched Ti_3_AlC_2_ powders can be successfully separated into Ti_3_C_2_T_x_ nanosheets under ultrasound treatment without using any intercalation agent. The relevant etching mechanism is may be attributed to the synergy effect of H_2_SO_4_ and H_2_O_2_, which triggers sequential selective etching of Al layers from the Ti_3_AlC_2_ phase. It may provide a new green way to prepare MXene-based materials without using toxic HF or HF-containing etchants.

## Introduction

Since a new family of two-dimensional (2D) materials was first discovered in 2011 (Naguib et al., 2011), increasing attention has been paid to these novel 2D transition metal carbides, carbonitrides, and nitrides (named MXenes), and currently, more than 40 MXene compositions (Naguib et al., 2021) have been synthesized by different efficient routes (Naguib and Gogotsi, 2015; Alhabeb et al., 2017; Anasori et al., 2017; Tao et al., 2017; Zhou et al., 2017; Gogotsi and Anasori, 2019; Fan et al., 2022). The MAX phases are layered ternary carbides and nitrides with a general formula M_n+1_AX_n_, where M represents transition metals (such as Sc, Ti, Zr, Hf, V, Nb, Ta, Cr, Mo, etc.), A represents elements from the group 13 and 14 of the periodic table, and X is carbon and/or nitrogen (Naguib and Gogotsi, 2015). After the discovery of Ti_3_C_2_T_x_ MXene in 2011 (Naguib et al., 2011), many MXenes were synthesized by selectively etching different precursors in liquid mediums such as HF, HF-containing, or HF-forming etchants; thus, these etching processes unavoidably caused some surface functional groups such as −O, −F, or −OH, marked as T_x_ in a general formula M_n+1_X_n_T_x_ for MXenes (Naguib and Gogotsi, 2015; Anasori et al., 2017; Tao et al., 2017; Zhou et al., 2017).

More importantly, MXenes and MXene-based composite materials have potential applications in various fields such as energy storage (Lukatskaya et al., 2013; Ghidiu et al., 2014; Liang et al., 2015; Wang et al., 2015; Luo et al., 2020; VahidMohammadi et al., 2021), flexible electronics (Xu et al., 2021a; Xu et al., 2021b; Shi et al., 2022a; Xu et al., 2022), electromagnetic shielding (Shahzad et al., 2016; Iqbal et al., 2020), catalysis (Seh et al., 2016; Liu et al., 2020), and water treatment (Ren et al., 2015) due to their unique physical and chemical properties (Halim et al., 2014; Dillon et al., 2016; Hantanasirisakul et al., 2016). In order to promote their potential applications, numerous research efforts have been carried out to explore the emerging etching methods and the stable storage of MXene, especially the large-scale preparing methods (Naguib and Gogotsi, 2015; Alhabeb et al., 2017; Gogotsi and Anasori, 2019; Fan et al., 2022).

In general, MXene nanosheets were synthesized by selectively etching MAX phases, as well as subsequent intercalation and delamination. In the early stage, the typical etching methods were dominated by HF etching methods (Naguib et al., 2011) and *in situ* HF-forming etching methods (Ghidiu et al., 2014; Halim et al., 2014). For example, Gogotsi et al. designed that the synthesis of MXene can be scaled up in a small-batch wet chemical etching process, and 1.0 g of the Ti_3_AlC_2_ powders was slowly peeled off in a system of HF and HCl for 24 h at 35°C (Shuck et al., 2020). Since 2017, fluorine-free etching methods such as electrochemical etching methods (Pang et al., 2019), alkali etching methods (Li et al., 2017; Li et al., 2018), molten salt etching methods (Li et al., 2019), halogen etching methods (Jawaid et al., 2021; Shi et al., 2021), and other methods (Ghazaly et al., 2021) were developed to prepare fluorine-free MXenes with different characteristics. For example, the electrochemical etching method (Pang et al., 2019) and alkali etching method (Li et al., 2018) can avoid the use of fluorinated etchants, and at the same time, the HF toxicity to the human body, corrosiveness, and harm to the environment can be effectively avoided. In 2019, a new method for MAX phase etching at a high temperature of 550°C was developed by using Lewis acid molten salt as an etching agent (Li et al., 2019). This method avoids the use of HF, HF-containing, or HF-forming etchants, but the high temperature is a necessary condition. In 2021, Song et al. developed a controllable HCl-hydrothermal etching strategy for Mo_2_CT_x_ MXenes based on DFT calculation (Wang et al., 2021). In this process, Mo_2_CT_x_ MXenes were prepared through a hydrothermal etching process with concentrated HCl in an autoclave at 120 or 140°C for 5 days. However, the high concentration, high temperature, and long etching time also impede its widespread use. More recently, Xiao et al. delicately exploited a low-temperature photo-Fenton strategy to fabricate F-free Ti_3_C_2_ with 95% high purity, and this work would play an important role in the F-free fabrication of MXene and synthesis of cathodes with excellent performance for flexible lithium-sulfur batteries (Liang et al., 2022).

In addition, the intercalation and delamination strategies of MXenes are mainly divided into organic intercalator delamination, inorganic intercalator delamination, and mechanical delamination (Mashtalir et al., 2013; Sang et al., 2016). The principle of intercalation and delamination is based on weakening the interlayer force (such as hydrogen bonding and van der Waals forces) among MXene nanosheets (Naguib et al., 2011; Alhabeb et al., 2017).

Although many successful synthetic routes and delamination routes of MXenes have been reported in the literature (Naguib et al., 2011; Alhabeb et al., 2017; Gogotsi and Anasori, 2019; Naguib et al., 2021; Fan et al., 2022), there are still a number of disadvantages such as the corrosion of etching agent to experimental equipment, harmful impact of reagents on the environment, and severe operating conditions. Therefore, it is highly desirable to develop a simple, safe, and efficient protocol for the synthesis of MXenes, and it is still a major challenge.

Inspired by the synthesis of MXenes through a controllable HCl-hydrothermal etching (Wang et al., 2021), a selective etching of MAX phase (Ti_3_SiC_2_) by using a solution of HF with oxidant (such as H_2_O_2_) (Alhabeb et al., 2018) and highly reactive radicals (HO^•^ and O_2_^•−^) weakening the Ti–Al bonds in the MAX phase (Liang et al., 2022), we design a facile method to produce Ti_3_C_2_T_x_ MXenes by etching Ti_3_AlC_2_ powders in a system of H_2_SO_4_ and H_2_O_2_. In this system, an aqueous dilute H_2_SO_4_ solution is not volatile nor toxic, and H_2_O_2_ is a green oxidant; thus, this experimental process is safe and convenient to conduct in a beaker. The experimental results confirmed that Ti_3_C_2_T_x_ nanosheets can be successfully obtained, and this method is safe, rapid, and efficient.

## Experimental section

### Materials

Ti_3_AlC_2_ powders (98 wt% purity, 200 mesh) were purchased from Shanghai Rohn Reagent Co., Ltd. Sulfuric acid (H_2_SO_4_, 18 mol/L), hydrogen peroxide (H_2_O_2_, 30%), and sodium hydroxide (NaOH, analytical grade) were purchased from the National Pharmaceutical Reagent Company. All chemicals were used without further purification. Deionized water (a resistance of 18 MΩ) made from a Milli-Q solvent system was used throughout all the experiments.

### Preparation of Ti_3_C_2_T_x_ Sheets

As a typical MAX phase, Ti_3_AlC_2_ powders were selected to be etched in a mixture of H_2_SO_4_ and H_2_O_2_ in our experiment.

1) Al layer etching. Typically, 2.0 g of Ti_3_AlC_2_ powders and 50 ml of H_2_SO_4_ solution (6 mol/L) were added into a beaker (200 ml) under electromagnetic stirring conditions. Then, 20 ml of H_2_O_2_ (30%) was slowly dropped into the abovementioned dispersion within 30 min under electromagnetic stirring conditions. A temperature-controlled water pot was used to control the temperature of 40 °C for this etching. Next, the obtained dispersion was still stirred at 40 °C for 30 min to further etch the Al layers from the Ti_3_AlC_2_ phase.
2) Sediment cleaning. After etching Al layers, the obtained dispersion was centrifuged at 3,000 rpm for 10 min with a high-speed centrifuge (Neofuge1600R) to obtain the sediment and recycle supernatant (including the etched Al layers and H_2_SO_4_ solution), respectively. The obtained sediment was cleaned with deionized water several times until the pH of the dispersion was close to 7. In addition, a nanocomposite of Al_2_O_3_ and TiO_2_ can be obtained from the supernatant by adding an appropriate amount of NaOH solution.
3) Ultrasonic stripping. A small amount of the obtained sediment was taken out and placed in a beaker with deionized water. No intercalation agent was required in this process. An ultrasonic cleaner was used to separate the obtained sediment in the beaker for 20 min to prepare Ti_3_C_2_T_x_ sheets with appropriate ultrasonic power (KQ-300GDV, frequency 40 kHz, output power 300 W, 50% amplitude). After ultrasonic treatment, the obtained aqueous solution of MXenes was filtered through a mixed cellulose ester microporous membrane using a water-circulating multi-purpose vacuum pump (SHB-III). Finally, the obtained sample was dried in a vacuum at 60°C for 24 h for characterizations.

### Characterizations

The microstructure of Ti_3_AlC_2_ powders and Ti_3_C_2_T_x_ sheets were detected by scanning electron microscopy (SEM, Hitachi S-4800) and transmission electron microscopy (TEM, JEM-2100). The X-ray diffractometer (XRD, Bruker D8 diffractometer), Fourier transform infrared spectroscopy (FTIR, Nicolet5700), and X-ray photoelectron spectroscopy (XPS, K-alpha1063) were used to analyze the etching process and the relevant etching mechanism of Ti_3_AlC_2_ powders.

## Results and discussion

### Structure analysis

**FIGURE 1:**
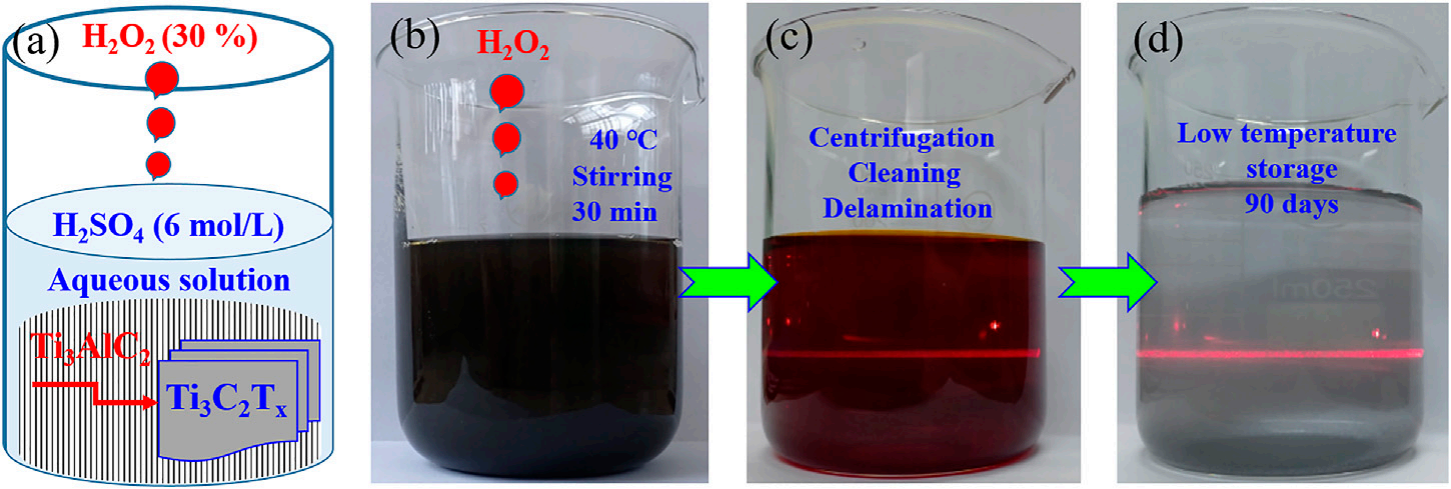
Ti_3_C_2_T_x_ nanosheets prepared by selective etching in a system of H_2_SO_4_ and H_2_O_2_. Schematic illustration of a preparation procedure in a beaker **(A)**. Digital photographs of a mixture of Ti_3_AlC_2_ powders and aqueous H_2_SO_4_ solution **(B)**, aqueous MXenes solution after Al layers etching **(C)**, and aqueous MXenes solution after delamination **(D)**.

Based on the synthetic strategies of HCl-hydrothermal etching (Wang et al., 2021), HF/H_2_O_2_ oxidant-assisted etching (Alhabeb et al., 2018), and photo-Fenton radicals weakening, (Liang et al., 2022) a facile, safe, and rapid strategy was designed to prepare Ti_3_C_2_T_x_ MXenes by selectively etching Ti_3_AlC_2_ powders in an aqueous H_2_SO_4_ solution with H_2_O_2_ as oxidant. The detailed preparation process (including etching, cleaning, and stripping) was introduced in the experimental section, and the schematic procedure is shown in Figure 1A. The mixture of Ti_3_AlC_2_ powders and aqueous H_2_SO_4_ solution is black (Figure 1B), while an aqueous solution of Ti_3_C_2_T_x_ sheets without cleaning and stripping has a purple/magenta color (Naguib et al., 2011), as shown in Figure 1C. After cleaning and stripping, the resulting aqueous solution of Ti_3_C_2_T_x_ nanosheets presents a shallow green color (Figure 1D). Both the aqueous solutions have clear Tyndall effects under natural light (Li et al., 2017), indicating the presence of Ti_3_C_2_T_x_ nanosheets in two aqueous solutions, as shown in Figures 1C,D. Moreover, the resulting aqueous solution of Ti_3_C_2_T_x_ nanosheets can be kept for 90 days under the low temperature of 1–4°C without any color change.

**FIGURE 2:**
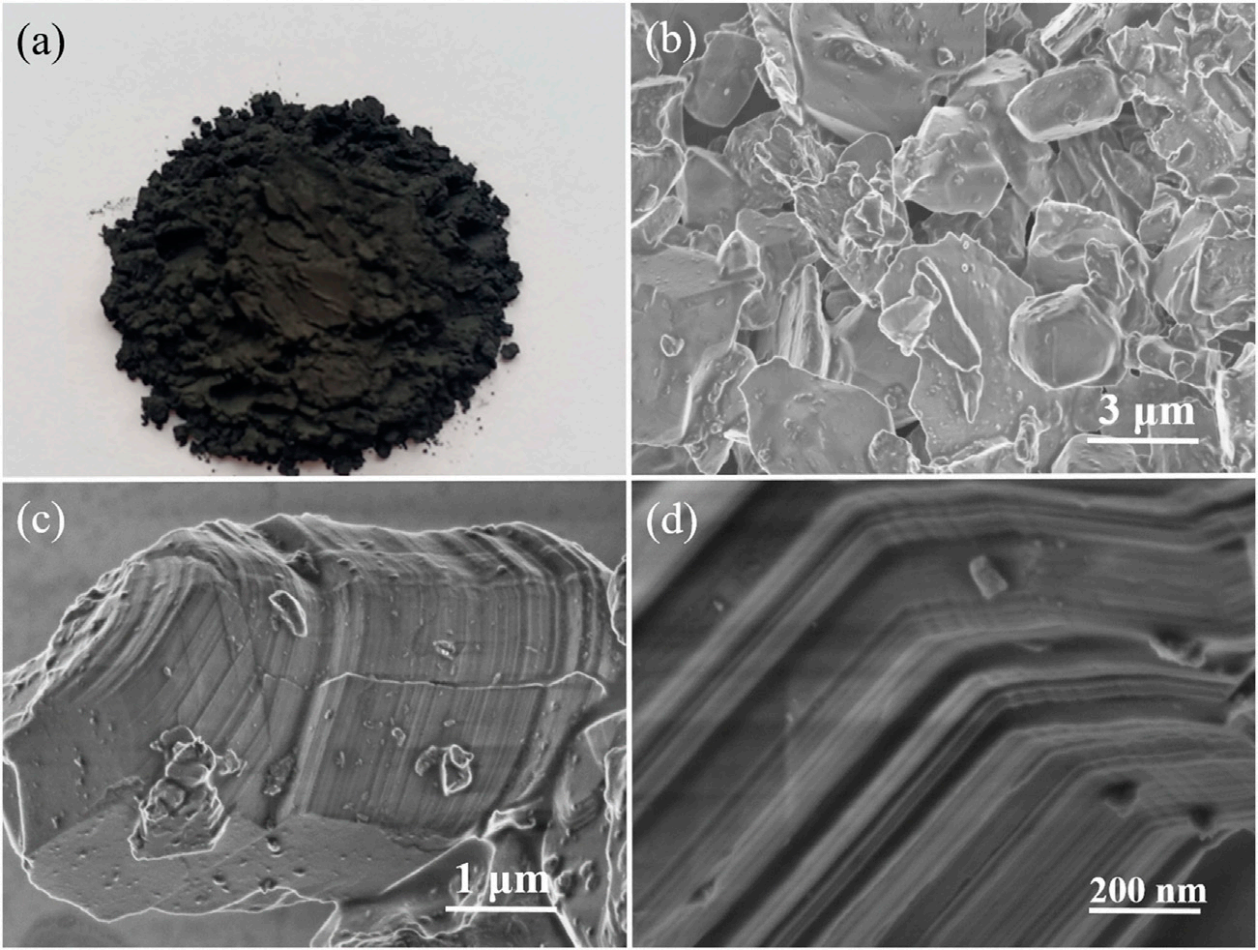
Digital photograph of the Ti_3_AlC_2_ powders **(A)**, SEM images of the Ti_3_AlC_2_ powders with different magnifications **(B–C)**, and magnified section SEM image of individual particle of Ti_3_AlC_2_ powders **(D)**.

In order to explore the etching effect, SEM images of the Ti_3_AlC_2_ powders and the obtained Ti_3_C_2_T_x_ sheets are carried out as contrast experiments. Figure 2 shows the microstructure of the Ti_3_AlC_2_ powders with different magnifications. It can be seen that the morphology and size of the Ti_3_AlC_2_ powders are very irregular (Figure 2B), and the individual particle has a compact layered structure, as shown in Figures 2C,D.

**FIGURE 3:**
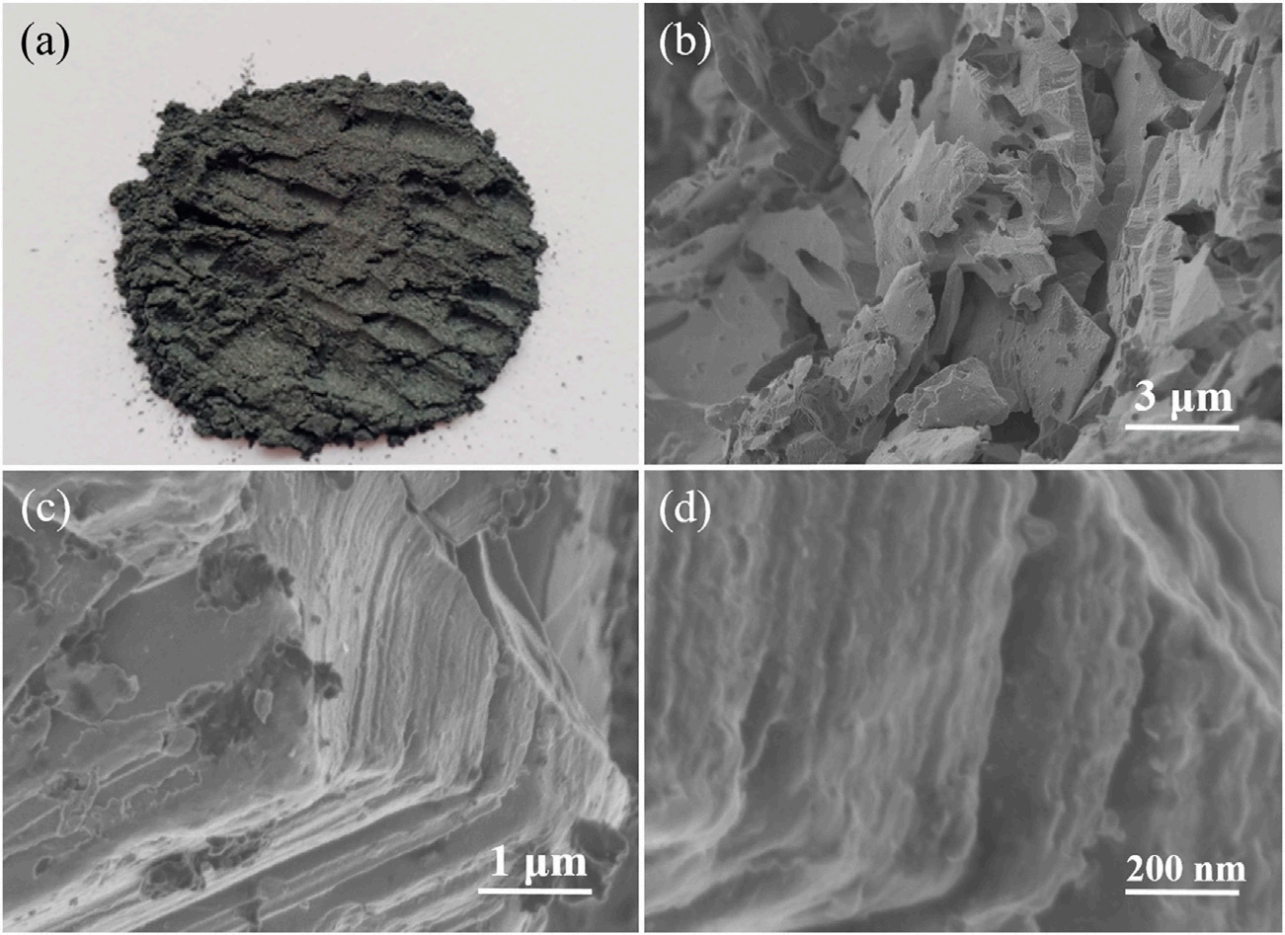
Digital photograph of Ti_3_C_2_T_x_ sheets **(A)**, SEM images of Ti_3_C_2_T_x_ sheets obtained by etching Ti_3_AlC_2_ powders in the H_2_SO_4_/H_2_O_2_ solution with different magnifications **(B–C)**, and magnified cross-section SEM image of Ti_3_C_2_T_x_ sheets **(D)**.

In contrast, SEM images in Figure 3 show the structural morphology of the obtained Ti_3_C_2_T_x_ sheets. The color of the obtained Ti_3_C_2_T_x_ MXene sheets is different from that of the Ti_3_AlC_2_ powders, namely, the colors change from black (Figure 2A) of the Ti_3_AlC_2_ powders to dark gray of the MXene sheets (Figure 3A). More importantly, the layered structure of the obtained Ti_3_C_2_T_x_ sheets is notably different from that of the Ti_3_AlC_2_ powders (Figures 2B–D), as clearly demonstrated in Figures 3B–D. Particularly, the structure of the obtained Ti_3_C_2_T_x_ sheets has some layered changes, and the lamellae have many hole defects, compared with that of the Ti_3_AlC_2_ powders; it is reasoned that when Al layers are etched away, a small amount of Ti layers is also etched out to leave holes and TiO_2_ nanoparticles on the surfaces due to the Ti vacancies triggering the oxidation process from Ti to TiO_2_, consistent with the results previous literature reported (Shi et al., 2022b; Huang et al., 2022; Jiang et al., 2022; Ma et al., 2022; Zhang et al., 2022).

**FIGURE 4:**
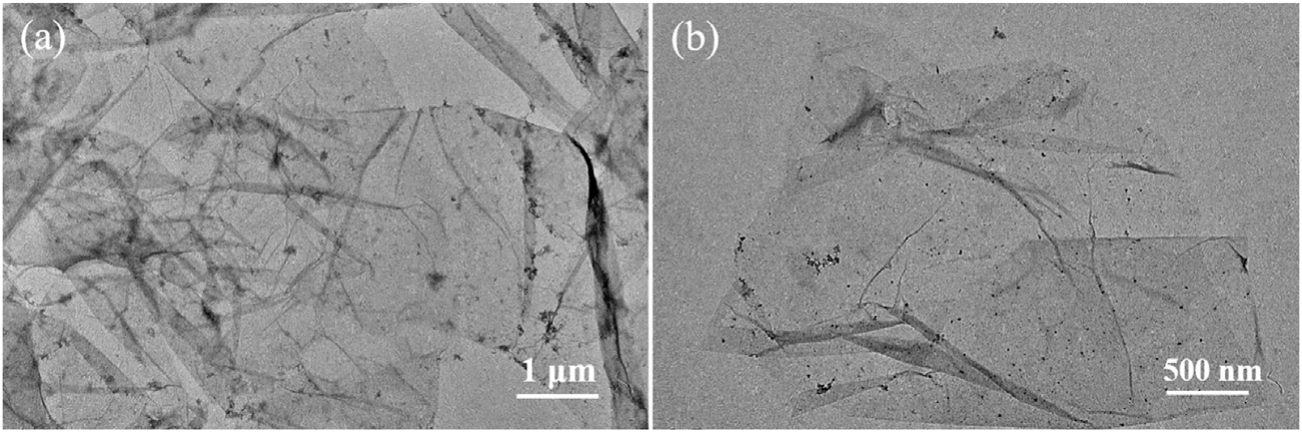
TEM images of Ti_3_C_2_T_x_ nanosheets with different magnifications **(A)** and **(B)**.

To further demonstrate the morphology of Ti_3_C_2_T_x_ sheets, TEM imaging was performed after the obtained Ti_3_C_2_T_x_ sheets were treated by ultrasonic stripping in deionized water for 20 min. As shown in Figure 4, the obtained Ti_3_C_2_T_x_ nanosheets are single-layer or multilayer sheets with folded structures, consistent with the reported literature (Huang et al., 2022; Ma et al., 2022). A slight amount of TiO_2_ nanoparticles on the MXene surface can be ascribed to the Ti vacancies triggering the oxidation process from Ti to TiO_2_, consistent with SEM images in Figures 3B–D. Thus, the Ti_3_AlC_2_ powders can be easily stripped into Ti_3_C_2_T_x_ nanosheets by a simple oxidative etching and subsequent ultrasonic exfoliation in our experiments.

**FIGURE 5:**
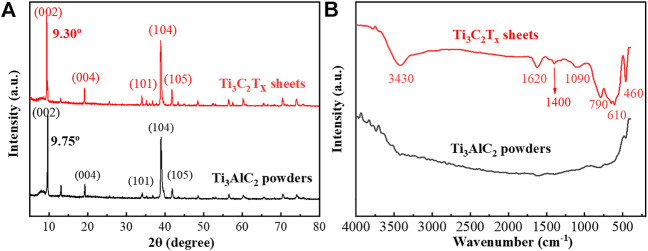
XRD patterns of Ti_3_AlC_2_ powders and Ti_3_C_2_T_x_ sheets **(A)**, and FTIR spectra of Ti_3_AlC_2_ powders and Ti_3_C_2_T_x_ sheets **(B)**.

In order to analyze the etching process, the XRD patterns and FTIR spectra of the Ti_3_AlC_2_ powders and the Ti_3_C_2_T_x_ sheets were used to monitor their structural changes. From the distribution of the XRD peaks in Figure 5A, the Ti_3_AlC_2_ powders and Ti_3_C_2_T_x_ sheets have characteristic peaks at different positions, such as (002), (004), (101), (104), (105), and so on (Naguib et al., 2011; Alhabeb et al., 2017; Li et al., 2017; Li et al., 2018; Li et al., 2019). In particular, the XRD (002) peak of the Ti_3_C_2_T_x_ sheets is shifted toward a lower angle, namely, the peaks change from 9.75° of Ti_3_AlC_2_ powders to 9.30° of Ti_3_C_2_T_x_ sheets, indicating a larger spacing caused by etching Al atoms. Moreover, a certain amount of Ti_3_AlC_2_ is probably still present in Ti_3_C_2_T_x_. From the FTIR spectra in Figure 5B, some new peaks appear, and they are very different from that of the pristine Ti_3_AlC_2_ powders. It can be found that there are lots of surface functional groups (Gao et al., 2021) such as 3,430 cm^−1^ of -OH, 1,620 cm^−1^ assigned to C=O, 1,400 cm^−1^ to the hydrogen bond of O-H, 1,090 cm^−1^ of C-O-C vibration, 790 cm^−1^ of the Ti-O bond (Wang et al., 2017), 610 cm^−1^ of SO_4_^2-^ vibration (Lin, 2015), and 460 cm^−1^ of Ti-C vibration (Wang et al., 2017). Thus, these new peaks of the FTIR spectrum may be attributed to the oxidation of H_2_O_2_ and the intercalation of SO_4_^2-^ (Lin, 2015; Wang et al., 2017; Gao et al., 2021).

**FIGURE 6:**
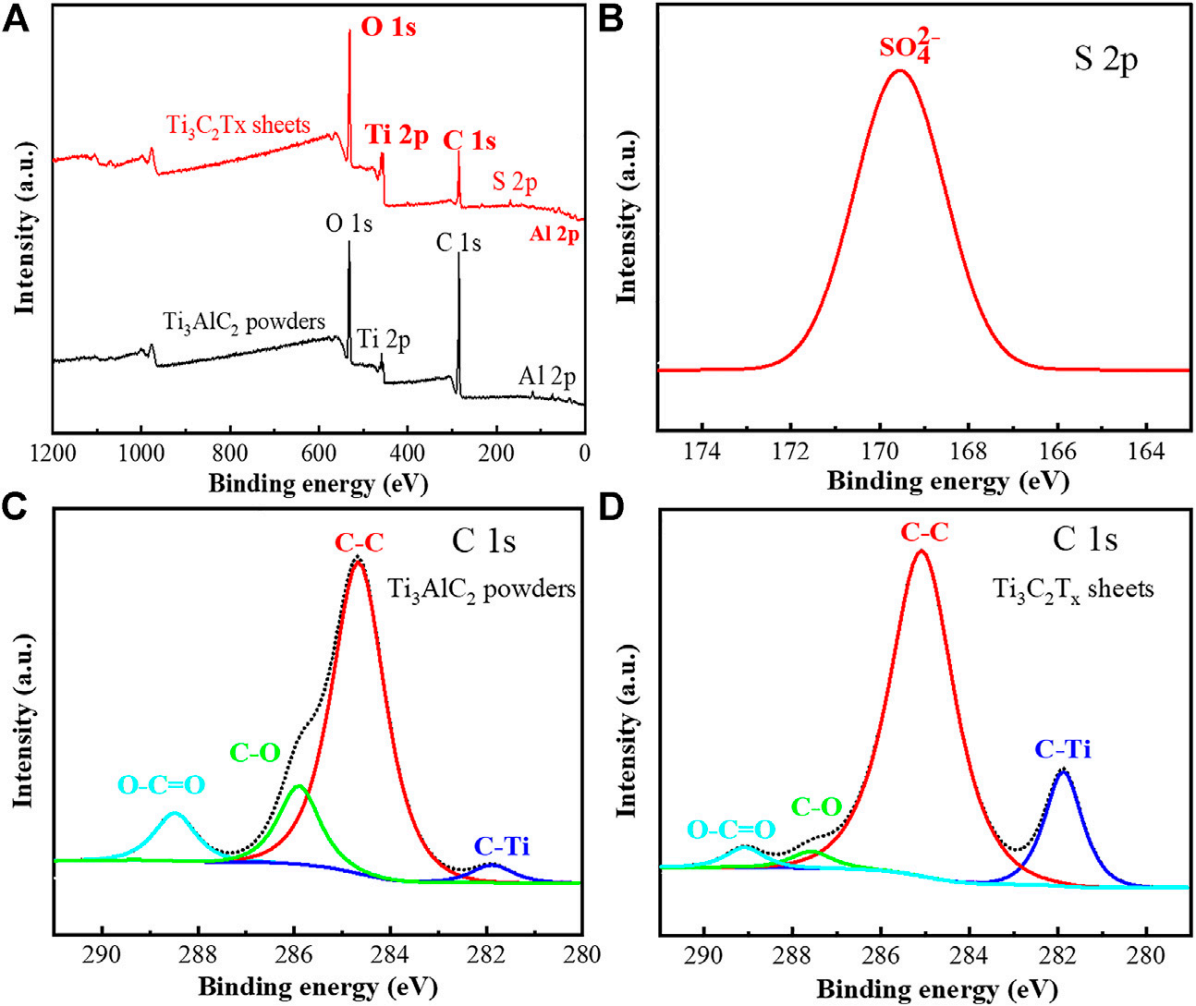
XPS survey spectra of Ti_3_AlC_2_ powders and Ti_3_C_2_T_x_ sheets **(A)**, high-resolution S 2p XPS spectrum of Ti_3_C_2_T_x_ sheets **(B)**, high-resolution C 1s XPS spectra of Ti_3_AlC_2_ powders and Ti_3_C_2_T_x_ sheets, respectively **(C)** and **(D)**.

In order to further explore the oxidation etching process, the XPS analysis of Ti_3_AlC_2_ powders and Ti_3_C_2_T_x_ sheets were performed to reveal their changes in chemical compositions. It can be found that the XPS survey spectra of Ti_3_C_2_T_x_ sheets present some new changes, such as a new peak of S 2p and a relatively elevated ratio of O 1s peak to C 1s peak (Figure 6A), compared with that of Ti_3_AlC_2_ powders.

Moreover, the high-resolution S 2p XPS analysis (Figure 6B) confirms that the new peak (Park and Leitao, 2021) of S 2p is attributed to SO_4_^2-^, consistent with the abovementioned FTIR analysis (Figure 5B). As shown in Figures 6C,D, the characteristic C 1s peaks of the Ti_3_AlC_2_ powders and Ti_3_C_2_T_x_ sheets also match those of previous work (Chen et al., 2020; Mathis et al., 2021), and the content ratio of C–Ti in the Ti_3_C_2_T_x_ sheets is higher than that of Ti_3_AlC_2_ powders, suggesting that most of Al atomic layers in Ti_3_C_2_T_x_ sheets have been selectively stripped.

**FIGURE 7:**
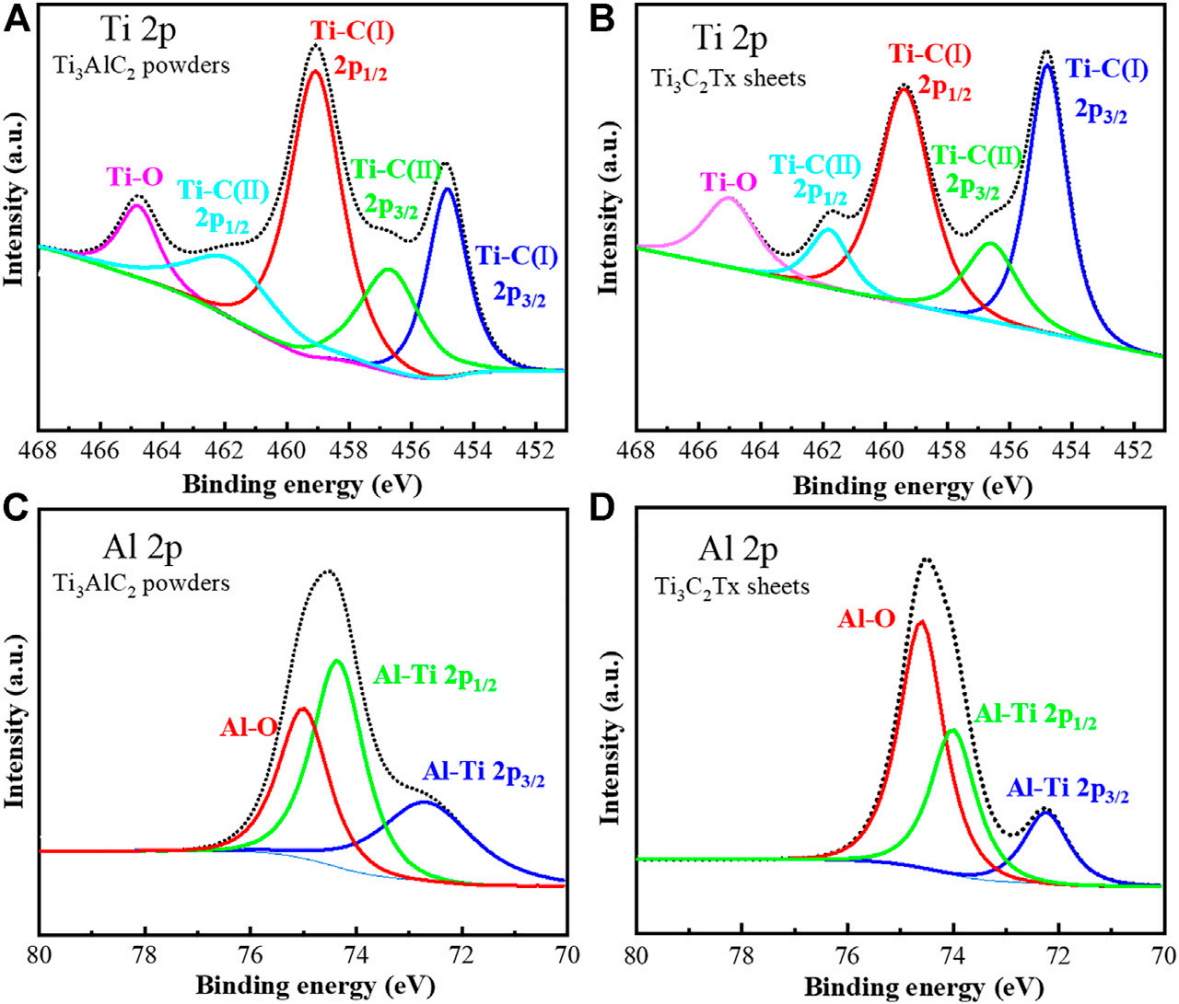
High-resolution XPS spectra of Ti 2p **(A)** and **(B)** and Al 2p **(C)** and **(D)** of Ti_3_AlC_2_ powders and Ti_3_C_2_T_x_ sheets, respectively.

The high-resolution Ti 2p XPS spectra of Ti_3_AlC_2_ powders and Ti_3_C_2_T_x_ sheets show three characteristic peaks, corresponding to the Ti-C 2p_3/2_, Ti-C 2p_1/2_, and Ti-O orbitals, respectively (Figures 7A,B), consistent with the current literature reports (Li et al., 2019; Chen et al., 2020; Mathis et al., 2021). Moreover, based on the analysis of XPS data, the content of Ti increases from 5.50 At% of Ti_3_AlC_2_ powders to 10.50 At% of Ti_3_C_2_T_x_ sheets.

On the other hand, the high-resolution Al 2p XPS spectra of Ti_3_AlC_2_ powders and Ti_3_C_2_T_x_ sheets show three characteristic peaks (Chen et al., 2020; Mathis et al., 2021) corresponding to the Al-Ti 2p_3/2_, Al-Ti 2p_1/2_, and Al-O orbitals, respectively (Figures 7C,D). In particular, the content ratio of Al-Ti 2p_3/2_ and Al-Ti 2p_1/2_ in the Ti_3_C_2_T_x_ sheets is significantly lower than that of Ti_3_AlC_2_ powders; in contrast, the content ratio of Al-O is higher. Based on the XPS analysis, the content of Al decreases from 6.50 At% of Ti_3_AlC_2_ powders to 1.50 At% of Ti_3_C_2_T_x_ sheets. Thus, from the abovementioned content changes of Ti and Al on the XPS analysis before and after corrosion, it can be reasoned that most of the Al layers can be etched out; as a result, the atomic percentage of Al decreases, and the atomic percentage of Ti increases in the Ti_3_C_2_T_x_ sheets.

### Mechanism analysis

**FIGURE 8:**
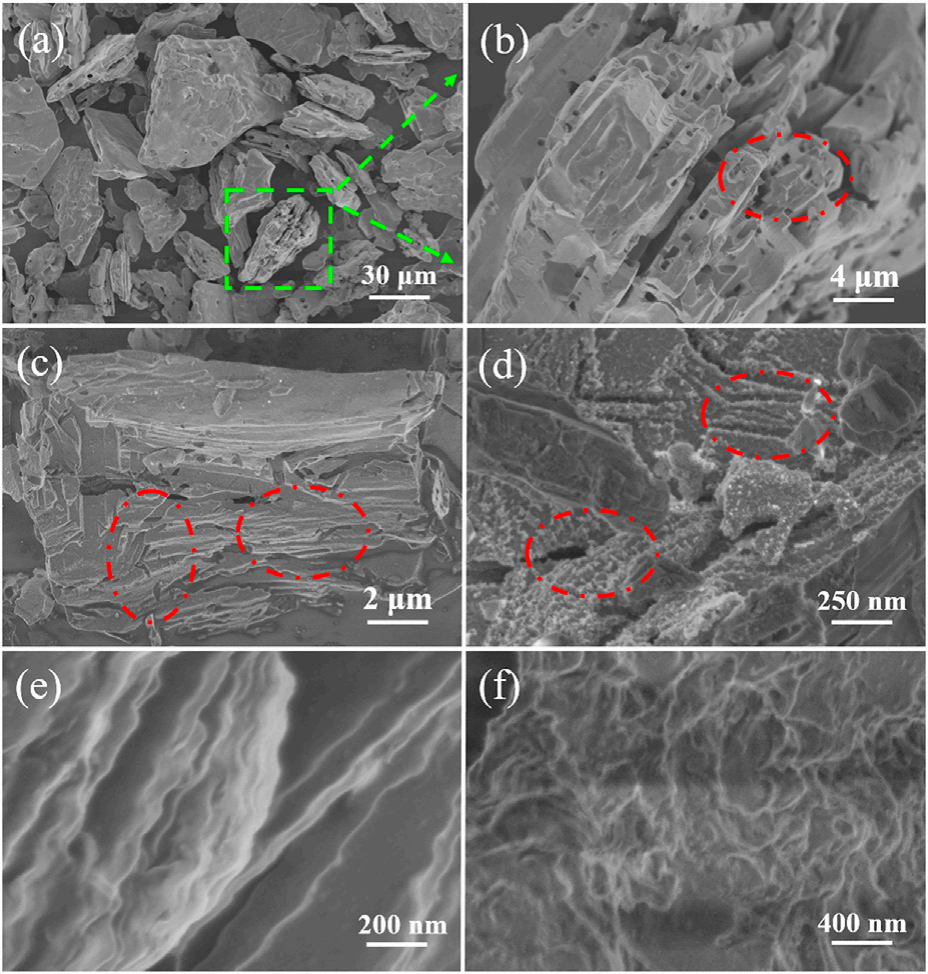
SEM images of intermediate sheets obtained by etching Ti_3_AlC_2_ powders in the H_2_SO_4_/H_2_O_2_ solution with different magnifications **(A–D)**, and SEM images of Ti_3_C_2_T_x_ nanosheets with different magnifications **(E–F)**.

To analyze the selective etching process, some important factors should be considered as follows:

1) In the system of H_2_SO_4_ and H_2_O_2_, the aqueous solution H_2_SO_4_ of 6 mol/L is a strongly acidic medium with no volatility and 30% H_2_O_2_ acts as a strong green oxidizer without harmful by-products.
2) As the temperature is an important factor for the chemical reaction, to speed up the etching process, the temperature of the obtained mixture is heated by a temperature-controlled water pot. In our experiment, if the temperature is too high, it will accelerate the decomposition of H_2_O_2_, and 40°C is chosen as the appropriate temperature. Moreover, no notable reaction occurs when the Ti_3_AlC_2_ powders are only mixed with an H_2_SO_4_ solution of 6 mol/L, and it is reasoned that the oxidant of H_2_O_2_ plays a major role in the etching process. Particularly, the content of H_2_O_2_ is very crucial for the etching process; if the oxidant of H_2_O_2_ is excessive, there will be some TiO_2_ nanoparticles on the surface and interspace of MXene sheets because Ti can be oxidized to TiO_2_ by excessive H_2_O_2_ (Huang et al., 2022; Ma et al., 2022). Otherwise, a certain amount of Ti_3_AlC_2_ is still present in Ti_3_C_2_T_x_ without corrosion.
3) In the etching process, the microstructure change of intermediate sheets should be analyzed in detail. Seen from SEM images in Figures 8A,B, the obtained intermediate sheets show a loose, layered, and porous structure compared with Ti_3_AlC_2_ powders. This structure can be clearly demonstrated by the magnified parts of the selected area of the intermediate sheets (Figure 8B). Moreover, Figures 8C,D further reveal that the intermediate sheets are exfoliated along different directions, and lots of traces after stripping are left on the surface such as crack structures along longitudinal/transverse direction and TiO_2_ nanoparticles on the surface (marked in the red circle of Figure 8D). Thus, the obtained Ti_3_C_2_T_x_ nanosheets with clear layered and folded structures can be easily prepared after the intermediate sheets are stripped apart under ultrasonic treatment in deionized water (Figures 8E,F).

**FIGURE 9:**
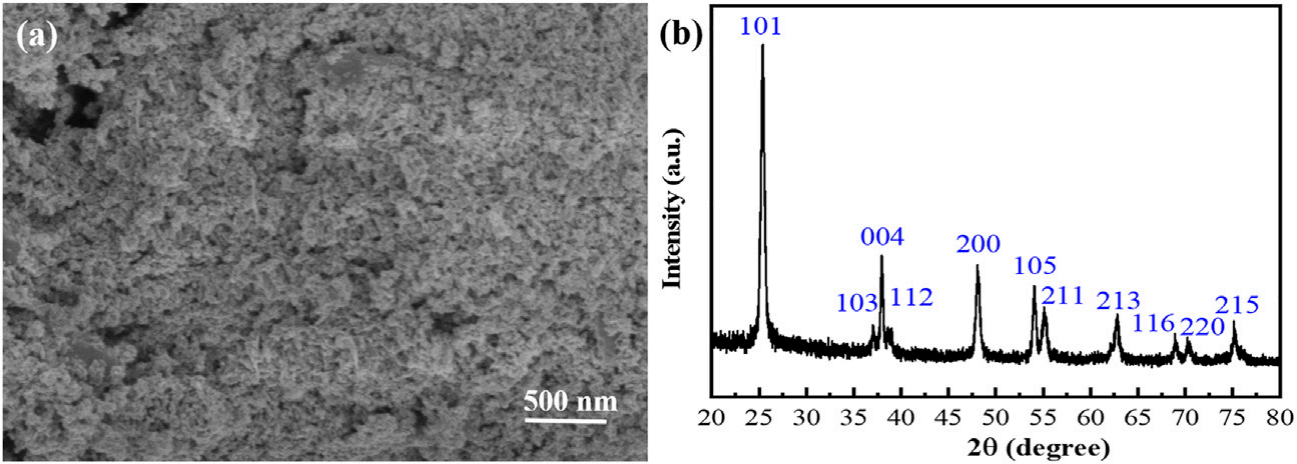
SEM image **(A)** and XRD patterns **(B)** of Al_2_O_3_ and TiO_2_ nanocomposite.

Moreover, the Al_2_O_3_ and TiO_2_ nanocomposite (Figure 9A) can be obtained from the supernatant by adding a certain amount of NaOH solution, vacuum filtration, and drying. The precipitate of Al_2_O_3_ and TiO_2_ can be precipitated by only adding an alkaline solution, which suggests that Al^3+^ ions accompanied by a small amount of TiO_2_ are in the supernatant. As shown in Figure 9B, the XRD patterns of this nanocomposite are analyzed to prove the existence of Al_2_O_3_ and TiO_2_ nanoparticles, consistent with those reported in the literature (Han et al., 2012; Abazari et al., 2014; Ali et al., 2018).

In summary, the relevant etching mechanism is may be attributed to the synergy effect of H_2_SO_4_ and H_2_O_2_, which triggers Al layers to be etched sequentially from the MAX phase. It is speculated that the reactive oxygen species (such as HO^•^ and O_2_^•-^) radicals can be produced because H_2_O_2_ is heated in the course of our experiment, very similar to the advanced oxidation process of the photo-Fenton reaction (Liang et al., 2022). Thus, the radicals can weaken the Ti–Al bonds and attack the defect sites of the external surfaces of MAX phases (Liu et al., 2020; Wei et al., 2021), and synchronously, Al layers are etched into Al^3+^ ions in the aqueous H_2_SO_4_ solution of 6 mol/L, and there is no accumulation of Al(OH)_3_ in this strongly acidic solution to retard this etching process. In addition, a lot of bubbles (such as O_2_ and H_2_) are produced to promote this etching process. Therefore, the synergy effect of H_2_SO_4_ and H_2_O_2_ triggers sequential selective etching of Al layers from the Ti_3_AlC_2_ phase.

On the other hand, abundant oxygen-containing functional groups are attached to the intermediate sheets, and Ti can be oxidized to TiO_2_ by excessive H_2_O_2_. Interestingly, the SO_4_^2-^ ions can be also inserted into the obtained Ti_3_C_2_T_x_ nanosheets and TiO_2_ nanoparticles can be *in situ* loaded on the surface of the obtained MXene nanosheets. The structure and morphology of the obtained MXene nanosheets can be further optimized by selecting the appropriate process such as the reaction rate and content of H_2_O_2_.

## Conclusion

In conclusion, we develop a simple, safe, and efficient method to prepare MXene (Ti_3_C_2_T_x_) nanosheets by selective etching Ti_3_AlC_2_ powders in a system of H_2_SO_4_/H_2_O_2_ and subsequent ultrasonic stripping. The obtained Ti_3_C_2_T_x_ nanosheets can be confirmed from the characterization analyses of SEM, TEM, XRD, FTIR, and XPS. Lots of oxygen-containing functional groups (−O, −OH) are supported on the MXene surface, the SO_4_^2-^ ions are inserted into the layers of the MXene sheets, and even TiO_2_ nanoparticles can be *in situ* loaded due to the oxidation of excessive H_2_O_2_. In the system of H_2_SO_4_/H_2_O_2_, the aqueous H_2_SO_4_ solution of 6 mol/L is a strongly acidic medium, and 30% H_2_O_2_ acts as a strong green oxidizer. The relevant etching mechanism may be attributed to the synergy effect of H_2_SO_4_ and H_2_O_2_, which triggers Al layers to be etched sequentially from the MAX phase. Our group is further exploring the etching mechanism. This work can provide a new way to develop HF-free and large-scale synthesis of 2D layered MXenes for many practical applications.

## Data Availability

The original contributions presented in the study are included in the article/Supplementary Material; further inquiries can be directed to the corresponding authors.

## References

[B1] Abazari R. Mahjoub A. R. Sanati S. (2014). A facile and efficient preparation of anatase titania nanoparticles in micelle nanoreactors: Morphology, structure, and their high photocatalytic activity under UV light illumination. RSC Adv. 4, 56406–56414. 10.1039/c4ra10018b

[B2] Alhabeb M. Maleski K. Anasori B. Lelyukh P. Clark L. Sin S. (2017). Guidelines for synthesis and processing of two-dimensional Titanium carbide (Ti_3_C_2_T_x_ MXene). Chem. Mat. 29, 7633–7644. 10.1021/acs.chemmater.7b02847

[B3] Alhabeb M. Maleski K. Mathis T. S. Sarycheva A. Hatter C. B. Uzun S. (2018). Selective etching of Silicon from Ti_3_SiC_2_ (MAX) to obtain 2D Titanium carbide (MXene). Angew. Chem. Int. Ed. Engl. 57, 5542–5546. 10.1002/ange.201802232 29518271

[B4] Ali A. S. Mohammed A. J. Saud H. R. (2018). Hydrothermal synthesis of TiO2/Al2O3 nanocomposite and its application as improved sonocatalyst. Int. J. Eng. Technol. 7, 22–25. 10.14419/ijet.v7i4.37.23607

[B5] Anasori B. Lukatskaya M. R. Gogotsi Y. (2017). 2D metal carbides and nitrides (MXenes) for energy storage. Nat. Rev. Mat. 2, 16098. 10.1038/natrevmats.2016.98

[B6] Chen W. Y. Jiang X. Lai S-N. Peroulis D. Stanciu L. (2020). Nanohybrids of a MXene and transition metal dichalcogenide for selective detection of volatile organic compounds. Nat. Commun. 11, 1302. 10.1038/s41467-020-15092-4 32157089 PMC7064528

[B7] Dillon A. D. Ghidiu M. J. Krick A. L. Griggs J. May S. J. Gogotsi Y. (2016). Highly conductive optical quality solution-processed films of 2D Titanium carbide. Adv. Funct. Mat. 26, 4162–4168. 10.1002/adfm.201600357

[B8] Fan Y. Li L. Zhang Y. Zhang X. Geng D. Hu W. (2022). Recent advances in growth of Transition metal carbides and nitrides (MXenes) crystals. Adv. Funct. Mat. 32, 2111357. 10.1002/adfm.202111357

[B9] Gao X. Wang B. Wang K. Xu S. Liu S. Liu X. (2021). Design of Ti3C2Tx/TiO2/PANI multi-layer composites for excellent electromagnetic wave absorption performance. J. Colloid Interface Sci. 583, 510–521. 10.1016/j.jcis.2020.09.094 33035791

[B10] Ghazaly A. E. Ahmed H. Rezk A. R. Halim J. Persson Per O. Å. Yeo L. Y. (2021). Ultrafast, one-step, salt-solution-based acoustic synthesis of Ti_3_C_2_ MXene. ACS Nano 15, 4287–4293. 10.1021/acsnano.0c07242 33635629 PMC8034768

[B11] Ghidiu M. Lukatskaya M. R. Zhao M. Q. Gogotsi Y. Barsoum M. W. (2014). Conductive two-dimensional Titanium carbide’clay’ with high volumetric capacitance. Nature 516, 78–81. 10.1038/nature13970 25470044

[B12] Gogotsi Y. Anasori B. (2019). The rise of MXenes. ACS Nano 13, 8491–8494. 10.1021/acsnano.9b06394 31454866

[B13] Halim J. Lukatskaya M. R. Cook K. M. Lu J. Smith C. R. Naslund L. A. (2014). Transparent conductive two-dimensional Titanium carbide epitaxial thin films. Chem. Mat. 26, 2374–2381. 10.1021/cm500641a PMC3982936 24741204

[B14] Han C. Luque R. Dionysiou D. D. (2012). Facile preparation of controllable size monodisperse anatase titania nanoparticles. Chem. Commun. 48, 1860–1862. 10.1039/c1cc16050h 22073398

[B15] Hantanasirisakul K. Zhao M. Q. Urbankowski P. Halim J. Anasori B. Kota S. (2016). Fabrication of Ti_3_C_2_T_x_ MXene transparent thin films with tunable optoelectronic properties. Adv. Electron. Mat. 2, 1600050. 10.1002/aelm.201600050

[B16] Huang Y. Lu Q. Wu D. Jiang Y. Liu Z. Chen B. (2022). Flexible MXene films for batteries and beyond. Carbon Energy 4, 598–620. 10.1002/cey2.200

[B17] Iqbal A. Shahzad F. Hantanasirisakul K. Kim M-K. Kwon J. Hong J. (2020). Anomalous absorption of electromagnetic waves by 2D transition metal carbonitride Ti_3_CNT_x_ (MXene). Science 369, 446–450. 10.1126/science.aba7977 32703878

[B18] Jawaid A. Hassan A. Neher G. Nepal D. Pachter R. Kennedy W. J. (2021). Halogen etch of Ti_3_AlC_2_ MAX phase for MXene fabrication. ACS Nano 15, 2771–2777. 10.1021/acsnano.0c08630 33502839

[B19] Jiang J. Bai S. Zou J. Liu S. Hsu J-P. Li N. (2022). Improving stability of MXenes. Nano Res. 15, 6551–6567. 10.1007/s12274-022-4312-8

[B20] Li G. Tan L. Zhang Y. Wu B. Li L. (2017). Highly efficiently delaminated single-layered MXene nanosheets with large lateral size. Langmuir 33, 9000–9006. 10.1021/acs.langmuir.7b01339 28805394

[B21] Li M. Lu J. Luo K. Li Y. Chang K. Chen K. (2019). Element replacement approach by reaction with Lewis acidic molten salts to synthesize nanolaminated MAX phases and MXenes. J. Am. Chem. Soc. 141, 4730–4737. 10.1021/jacs.9b00574 30821963

[B22] Li T. Yao L. Liu Q. Gu J. Luo R. Li J. (2018). Fluorine-free synthesis of high-purity Ti_3_C_2_T_x_ (T= OH, O) *via* alkali treatment. Angew. Chem. Int. Ed. 57, 6115–6119. 10.1002/anie.201800887 29633442

[B23] Liang L. Niu L. Wu T. Zhou D. Xiao Z. (2022). Fluorine-free fabrication of MXene *via* photo-Fenton approach for advanced lithium-sulfur batteries. ACS Nano 16, 7971–7981. 10.1021/acsnano.2c00779 35466669

[B24] Liang X. Garsuch A. Nazar L. F. (2015). Sulfur cathodes based on conductive MXene nanosheets for high-performance lithium-sulfur batteries. Angew. Chem. Int. Ed. Engl. 54, 3979–3983. 10.1002/ange.201410174 25650042

[B25] Lin X. H. (2015). Impact of the spatial distribution of sulfate species on the activities of SO42−/TiO2 photocatalysts for the degradation of organic pollutants in reverse osmosis concentrate. Appl. Catal. B Environ. 170, 263–272. 10.1016/j.apcatb.2015.02.001

[B26] Liu A. Liang X. Ren X. Guan W. Gao M. Yang Y. (2020). Recent progress in MXene-based materials: Potential high-performance electrocatalysts. Adv. Funct. Mat. 30, 2003437. 10.1002/adfm.202003437

[B27] Lukatskaya M. R. Mashtalir O. Ren C. E. Dall’Agnese Y. Rozier P. Taberna P. L. (2013). Cation intercalation and high volumetric capacitance of two-dimensional Titanium carbide. Science 341, 1502–1505. 10.1126/science.1241488 24072919

[B28] Luo J. Matios E. Wang H. Tao X. Li W. (2020). Interfacial structure design of MXene-based nanomaterials for electrochemical energy storage and conversion. InfoMat 2, 1057–1076. 10.1002/inf2.12118

[B29] Ma Y. Cheng Y. Wang J. Fu S. Zhou M. Yang Y. (2022). Flexible and highly-sensitive pressure sensor based on controllably oxidized MXene. InfoMat 4, e12328. 10.1002/inf2.12328

[B30] Mashtalir O. Naguib M. Mochalin V. N. Dall’Agnese Y. Heon M. Barsoum M. W. (2013). Intercalation and delamination of layered carbides and carbonitrides. Nat. Commun. 4, 1716. 10.1038/ncomms2664 23591883

[B31] Mathis T. S. Maleski K. Goad A. Sarycheva A. Anayee M. Foucher A. C. (2021). Modified MAX phase synthesis for environmentally stable and highly conductive Ti3C2 MXene. ACS Nano 15, 6420–6429. 10.1021/acsnano.0c08357 33848136

[B32] Naguib M. Barsoum M. W. Gogotsi Y. (2021). Ten years of progress in the synthesis and development of MXenes. Adv. Mat. 2021, 2103393. 10.1002/adma.202103393 34396592

[B33] Naguib M. Gogotsi Y. (2015). Synthesis of two-dimensional materials by selective extraction. Acc. Chem. Res. 48, 128–135. 10.1021/ar500346b 25489991

[B34] Naguib M. Kurtoglu M. Presser V. Lu J. Niu J. Heon M. (2011). Two-dimensional nanocrystals produced by exfoliation of Ti_3_AlC_2_ . Adv. Mat. 23, 4248–4253. 10.1002/adma.201102306 21861270

[B35] Pang S-Y. Wong Y-T. Yuan S. Liu Y. Tsang M-K. Yang Z. (2019). Universal strategy for HF-free facile and rapid synthesis of two-dimensional MXenes as multifunctional energy materials. J. Am. Chem. Soc. 141, 9610–9616. 10.1021/jacs.9b02578 31117483

[B36] Park K. W. Leitao E. M. (2021). The link to polysulfides and their applications. Chem. Commun. 57, 3190–3202. 10.1039/d1cc00505g 33720262

[B37] Ren C. E. Hatzell K. B. Alhabeb M. Ling Z. Mahmoud K. A. Gogotsi Y. (2015). Charge- and size-selective ion sieving through Ti_3_C_2_T_x_ MXene membranes. J. Phys. Chem. Lett. 6, 4026–4031. 10.1021/acs.jpclett.5b01895 26722772

[B38] Sang X. Xie Y. Lin M. W. Alhabeb M. Van Aken K. L. Gogotsi Y. (2016). Atomic defects in monolayer Titanium carbide (Ti_3_C_2_T_x_ ) MXene. ACS Nano 10, 9193–9200. 10.1021/acsnano.6b05240 27598326

[B39] Seh Z. W. Fredrickson K. D. Anasori B. Kibsgaard J. Strickler A. L. Lukatskaya M. R. (2016). Two-dimensional Molybdenum carbide (MXene) as an efficient electrocatalyst for hydrogen evolution. ACS Energy Lett. 1, 589–594. 10.1021/acsenergylett.6b00247

[B40] Shahzad F. Alhabeb M. Hatter C. B. Anasori B. Man Hong S. Koo C. M. (2016). Electromagnetic interference shielding with 2D transition metal carbides (MXenes). Science 353, 1137–1140. 10.1126/science.aag2421 27609888

[B41] Shi H. Zhang P. Liu Z. Park S. W. Lohe M. R. Wu Y. (2021). Ambient-stable two-dimensional Titanium carbide (MXene) enabled by Iodine etching. Angew. Chem. Int. Ed. Engl. 60, 8771–8775. 10.1002/ange.202015627 PMC8048443 33484049

[B42] Shi L-N. Cui L-T. Ji Y-R. Xie Y. Zhu Y-R. Yi T-F. (2022). Towards high-performance electrocatalysts: Activity optimization strategy of 2D MXenes-based nanomaterials for water-splitting. Coord. Chem. Rev. 469, 214668. 10.1016/j.ccr.2022.214668

[B43] Shi Y. Zhou D. Wu T. Xiao Z. (2022). Deciphering the Sb_4_O_5_Cl_2_-MXene hybrid as a potential anode material for advanced potassium-ion batteries. ACS Appl. Mat. Interfaces 14, 29905–29915. 10.1021/acsami.2c06948 35737889

[B44] Shuck C. E. Sarycheva A. Anayee M. Levitt A. Zhu Y. Uzun S. (2020). Scalable synthesis of Ti_3_C_2_Tx MXene. Adv. Eng. Mat. 2020, 1901241. 10.1002/adem.201901241

[B45] Tao Q. Dahlqvist M. Lu J. Kota S. Meshkian R. Halim J. (2017). Two-dimensional Mo_1.33_C MXene with divacancy ordering prepared from parent 3D laminate with in-plane chemical ordering. Nat. Commun. 8, 14949. 10.1038/ncomms14949 28440271 PMC5413966

[B46] VahidMohammadi A. Rosen J. Gogotsi Y. (2021). The world of two-dimensional carbides and nitrides (MXenes). Science 372, eabf1581. 10.1126/science.abf1581 34112665

[B47] Wang C. Shou H. Chen S. Wei S. Lin Y. Zhang P. (2021). HCl-based hydrothermal etching strategy toward fluoride-free MXenes. Adv. Mat. 33, 2101015. 10.1002/adma.202101015 34057261

[B48] Wang X. Kajiyama S. Iinuma H. Hosono E. Oro S. Moriguchi I. (2015). Pseudocapacitance of MXene nanosheets for high-power sodium-ion hybrid capacitors. Nat. Commun. 6, 6544. 10.1038/ncomms7544 25832913 PMC4396360

[B49] Wang Z. Xuan J. Zhao Z. Li Q. Geng F. (2017). Versatile cutting method for producing fluorescent ultrasmall MXene sheets. ACS Nano 11, 11559–11565. 10.1021/acsnano.7b06476 29111669

[B50] Wei Y. Zhang P. Soomro R. A. Zhu Q. Xu B. (2021). Advances in the synthesis of 2D MXenes. Adv. Mat. 2021, 2103148. 10.1002/adma.202103148 34423479

[B51] Xu M. Liang L. Qi J. Wu T. Zhou D. Xiao Z. (2021). Intralayered ostwald ripening‐induced self‐catalyzed growth of CNTs on MXene for robust lithium–sulfur batteries. Small 17, 2007446. 10.1002/smll.202007446 33733628

[B52] Xu M. Wu T. Qi J. Zhou D. Xiao Z. (2021). V_2_C/VO_2_ nanoribbon intertwined nanosheet dual heterostructure for highly flexible and robust lithium-sulfur batteries. J. Mat. Chem. A 9, 21429–21439. 10.1039/d1ta05693j

[B53] Xu M. Zhou D. Wu T. Qi J. Du Q. Xiao Z. (2022). Self-regulation of spin polarization density propelling the ion diffusion kinetics for flexible potassium-ion batteries. Adv. Funct. Mat. 2022, 2203263. 10.1002/adfm.202203263

[B54] Zhang M. Yang D. Dong C. Huang H. Feng G. Chen Q. (2022). Two-dimensional MXene-originated *in situ* nanosonosensitizer generation for augmented and synergistic sonodynamic tumor nanotherapy. ACS Nano 16, 9938–9952. 10.1021/acsnano.2c04630 35639357

[B55] Zhou J. Zha X. Zhou X. Chen F. Gao G. Wang S. (2017). Synthesis and electrochemical properties of two-dimensional Hafnium carbide. ACS Nano 11, 3841–3850. 10.1021/acsnano.7b00030 28375599

